# Statistical Investigation of the Mechanical and Geometrical Properties of Polysilicon Films through On-Chip Tests

**DOI:** 10.3390/mi9020053

**Published:** 2018-01-30

**Authors:** Ramin Mirzazadeh, Aldo Ghisi, Stefano Mariani

**Affiliations:** Dipartimento di Ingegneria Civile e Ambientale, Politecnico di Milano, Piazza Leonardo da Vinci 32, 20133 Milano, Italy; ramin.mirzazadeh@polimi.it (R.M.); stefano.mariani@polimi.it (S.M.)

**Keywords:** polysilicon morphology, over-etch, sensitivity to imperfections

## Abstract

In this work, we provide a numerical/experimental investigation of the micromechanics-induced scattered response of a polysilicon on-chip MEMS testing device, whose moving structure is constituted by a slender cantilever supporting a massive perforated plate. The geometry of the cantilever was specifically designed to emphasize the micromechanical effects, in compliance with the process constraints. To assess the effects of the variability of polysilicon morphology and of geometrical imperfections on the experimentally observed nonlinear sensor response, we adopt statistical Monte Carlo analyses resting on a coupled electromechanical finite element model of the device. For each analysis, the polysilicon morphology was digitally built through a Voronoi tessellation of the moving structure, whose geometry was in turn varied by sampling out of a uniform probability density function the value of the over-etch, considered as the main source of geometrical imperfections. The comparison between the statistics of numerical and experimental results is adopted to assess the relative significance of the uncertainties linked to variations in the micro-fabrication process, and the mechanical film properties due to the polysilicon morphology.

## 1. Introduction

Whenever we expose different micro-electromechanical systems (MEMS) devices, even taken from the same wafer, to the same input, different outcomes are likely to be observed. If the scattering in the measured response is not negligible, or higher than some thresholds defined for each specific application, remedies need to be envisaged. Although conceptually similar to macro scale problems, at the micro scale, the effect of uncertainties (especially those induced by the microstructural morphology and by the fabrication process) on the device performance or robustness can exponentially grow, as the dimensions of the device shrink. Concerning inertial MEMS sensors, two main sources of uncertainty and scattering can be identified: the geometry variation, and the fluctuation of the mechanical properties of movable structure(s). The geometrical and mechanical properties are often dealt with deterministically during the design stage; alternatively, they are handled as stochastic variables in a design for reliability context [1] using Monte Carlo or other methodologies, such as interval analysis [2] and approaches based on random variables [3]. Analysis of inaccuracies and parametric estimation [4,5] have been also sometimes adopted to capture and possibly reduce the influence of inaccuracies on some critical output parameters.

Narrowing the uncertainty intervals is not, however, always a feasible or economical solution, especially for consumer market oriented MEMS. For example, for silicon tuning fork commercial gyroscopes, it was shown in [6] that, in order to obtain a sense-drive frequency separation accuracy on the order of 2%, fabrication tolerances for critical suspension springs had to be close to the limit of the deep reactive-ion etching (DRIE) technology. Similarly, in [7,8], it was shown that, in order to make monolithically integrated microtensile testers, the role of uncertainties in the stiffness of critical MEMS components can become considerable.

In several MEMS applications, the mechanical components of the devices get deformed for actuation or sensing purposes: their stiffness represents therefore a key factor to characterize the behavior. In other words, either in statics or dynamics, the final device performance strongly depends on how close the actual and target values of the stiffness of the deformable components are; this issue gets more and more critical as the characteristic size of the components shrinks according to a miniaturization pathway. To ensure the efficiency of the devices in their working conditions, some ideas have been developed to adjust the stiffness after fabrication. A recent review on those methods can be found in [9], where the approaches were classified on the basis of the physical parameters they aim to modify, e.g., electrostatic forces, moment of inertia or Young’s modulus. Nevertheless, these workarounds can affect the performance indices of the device and, therefore, a thorough understanding of the origins and a quantitative assessment of the sources of uncertainty at the micro-scale are of crucial importance. In this work, we specifically focus on the statistical assessment of the stiffness variation of polysilicon films, as originated from uncertainties linked to material properties and geometry: while the former source has intrinsic origins, the latter one strongly depends on the micro-fabrication process, see e.g., [10,11].

As far as the investigation of the effects of the polycrystalline film properties on device performance is concerned, a wide range of micromechanical tests have been developed in the literature [12,13,14]. Published results reported an effective Young’s modulus of polysilicon, considered as an isotropic material in spite of its fcc crystal structure [15], in the range of 100–173 GPa, with a scattering of up to 15% [16,17,18,19,20,21,22]. Although the difference among the reported values can be explained through the dependency on the fabrication process, some of the studies showed a greater scattering for specimens featuring a smaller characteristic size [17,19]. Besides possible measurement errors, such variability can be associated to the statistical representativeness of the tested polysilicon volume (see [23,24,25]). If a few grains only can be found along the length of the tested micro-sized specimens, the hypothesis of (in-plane) isotropy does not hold true anymore for the film, as lattice grain orientation cannot be considered a random variable with a uniform distribution; as a consequence, the measured effective Young’s modulus can feature a large standard deviation [26,27]. In [23], for a material domain including less than 15×15=225 grains of a columnar film, it was shown that the scattering in the measured value cannot be less than 5%. In [28], the response of polysilicon films with a varying microstructure was investigated under tensile loading: it was reported that the relevant effective stiffness was independent of the film microstructure and of the grain size, provided that the sample dimensions guarantee statistical representativeness.

As far as geometrical uncertainties are instead concerned, slight variations from the target layout geometry are mainly due to scattering in the extent of acid attack, etching, and other common MEMS fabrication processes [29]. The resulting variability of the so-called over-etch (also known as over-cut) can be on the order of 0.1 ÷ 0.2 μm. Such over-etch is generally assumed to be uniform all over the lateral surfaces of a moving structure. However, for MEMS critical components such as slender supporting beams (or springs), the film stiffness could be heavily influenced by the over-etch altering their width. Over-etch has an impact not only on the beam stiffness but also on the capacitive gaps, and so it modifies both the electromechanical actuation and sensing properties of the device. Estimating the over-etch variability can be very difficult through direct visual observation techniques, since the estimation itself may depend on location of the measures. Such visual inspection, which is possible only for unpackaged devices, might also result to be an extremely time-consuming task.

Allowing for all the issues discussed here above, in this work, we propose a methodology to investigate the statistics of the micromechanics-induced scattering; we use an on-chip technique to deform a polysilicon microcantilever specimen featuring a few grains only across its width. Unlike other conventional techniques to investigate uncertainties at the micro-scale, the proposed device avoids using special read-out apparatuses, such as atomic force microscopy or laser Doppler vibrometry [4,23,30,31]. The device exploits electrostatic actuation and sensing, and does not require the wafer or the die to be open, with the movable structure being unprotected from the ambient environment. The outcomes of the experiments are interpreted through Monte Carlo analyses based on a numerical model of the whole device, in order to allow for parametric variations of its material properties and geometry. By adopting two different microcantilever lengths, we also provide experimental evidence of uncertainty intensification in the response of the whole device when the deformable components scale down close to the grain size. In former studies, see [32,33], the same device was adopted to identify the specimen-dependent characteristic values for the overall stiffness of the beam and for the over-etch. Those estimations were obtained by making use of an analytical model of the movable structure, within which the morphology of the polysilicon film was totally disregarded and only its effective properties were allowed for. As shown in [24], for polycrystalline geometries like the one here dealt with, see also [26], by accounting for the ratio between in-plane film width and the characteristic size of the silicon grains, information on the induced scattering of the microcantilever stiffness can be obtained. Anyway, important additional information on the effect of cantilever slenderness (provided by the length/width ratio) and on the type of deformation induced by the actuation, is somehow lost. To account for all the aforementioned features, and to also allow for the joint statistical effects of film morphology and over-etch depth, a two-dimensional finite element model of the movable structure is exploited in this work. Each silicon grain was explicitly modelled thanks to a Voronoi tessellation of the whole body; the displacement-induced distortion of the electrical domain was also accounted for to appropriately capture relevant response nonlinearities.

The remainder of this paper is organized as follows. In Section 2, the testing device and the experimental set-up are described, and the measured electromechanical responses are reported. Section 3 deals with the numerical modelling of the coupled electromechanical problem, with special focus on the stochastic representation of the polysilicon specimen. Results of Monte Carlo simulations are discussed in Section 4, where they are also compared with the experimental data. Finally, Section 5 provides some concluding remarks and open questions for future investigations.

## 2. Experimental: On-Chip Tests

**Figure 1:**
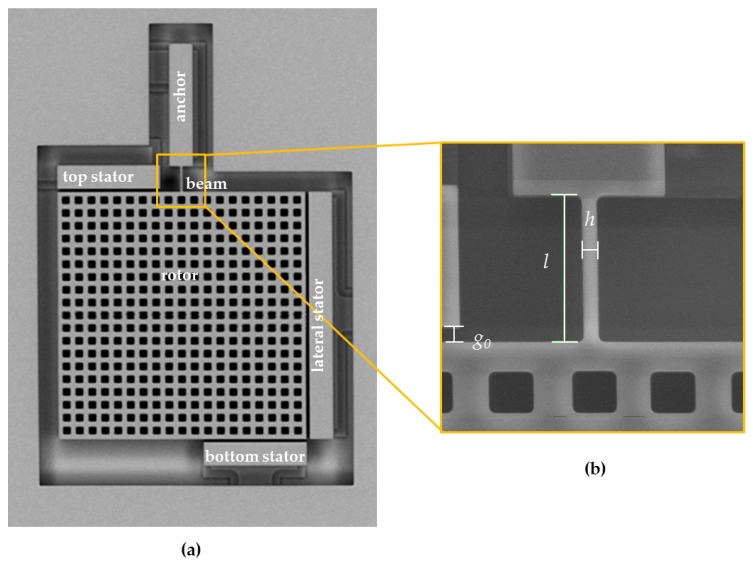
(**a**) SEM picture of the test device and (**b**) detail of the cantilever beam sample.

Experimental measurements were carried out on MEMS devices, whose movable parts are made of an epitaxially grown polysilicon film with an average grain size of 0.5 μm [34]. The device geometry is shown in Figure 1: the moving structure consists of a 200 μm × 200 μm perforated plate (labeled as rotor) connected on its top side to the anchor through a slender cantilever beam. Such statically determinate configuration was devised to reduce to a minimum the effect of residual stresses and stress gradients on the measured response. The beam nominal dimensions are: length *l* = 10 μm or 20 μm, out-of-plane thickness *t* = 22 μm, and width *h* = 2 μm.

Electrostatic actuation and sensing were respectively adopted to induce the motion and sense it through the stators placed around the rotor; for all the parallel plate capacitors, the nominal capacitance gap $g_{0}$ was set to 2 μm.

**Figure 2:**
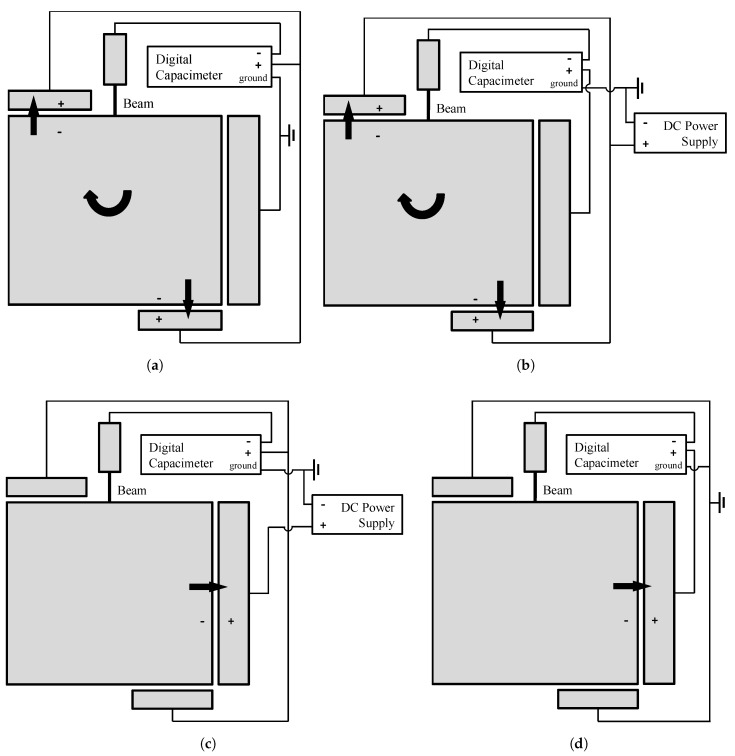
Adopted experimental set-ups: (**a**) RR configuration (rotational actuation and sensing); (**b**) RL configuration (rotational actuation, lateral sensing); (**c**) LR configuration (lateral actuation, rotational sensing); and (**d**) LL configuration (lateral actuation and sensing).

To define the actuation/sensing configuration, the following notation is adopted: a first index denotes the actuation type (either L or R), while a second index denotes the sensing type (again, either L or R). The four possible combinations of actuation and sensing are all reported in Figure 2. In these sketches and in what follows: L stands for lateral and means that the lateral stator is used for actuation and/or sensing; R stands instead for rotational and means that the top and bottom stators are both used to induce a rotation (only) of the rotor, or to sense the relevant effect. As depicted in Figure 2, two alternative set-ups were adopted to carry out the tests. With the first configuration (see Figure 2a,d), the capacimeter applied a DC voltage difference at one capacitor set (either L or R), and also measured the induced capacitance change. The resulting *C*–*V* curves (actually ΔC–*V*, where ΔC is the capacitance change with respect to the value corresponding to the configuration at rest V=0) increase monotonically, since the electrostatic forces induce a (rigid body) motion of the rotor, leading to an overall decrease of the capacitance gap. With the second configuration (see Figure 2b,c), a DC power supply provided the signal at one of the capacitor sets, while the capacimeter measured the change in the capacitance due to the rotor motion. In this latter case, the resulting *C*–*V* curves decrease monotonically, as the rotor motion provides an overall increase of the gap at the probed capacitor.

**Figure 3:**
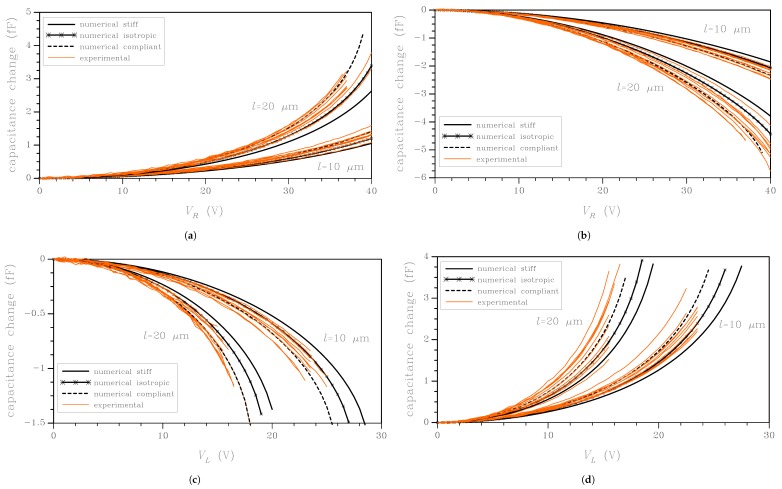
Experimental data and comparison with numerical results obtained using bounding and reference isotropic values for the film stiffness: (**a**) RR; (**b**) RL; (**c**) LR; and (**d**) LL test configurations.

The four test set-ups were devised to have two different actuation mechanisms and induce different stress/strain states in each cantilever beam: in the L case, a coupled bending-shear deformation mode was triggered; in the R case, a pure bending all over the beam length was instead induced. The two sensing mechanisms were allowed for to handle two sets of output data at assigned actuation, so to provide some redundancy in the observables if beam stiffness and over-etch have to be estimated, see [32,35,36]. To better distinguish the effects of film morphology and over-etch on measured *C*–*V* responses, different beam-lengths were considered too. Figure 3 gather the experimental *C*–*V* curves for the devices featuring both the values of the beam length *l* mentioned above. These plots also provide some reference, deterministic-like responses of the devices, computed as discussed in detail in Section 3. For each beam length, ten devices were tested using the four configurations. Each curve relevant to a specimen was obtained by repeating the test at least 30 times, up to a voltage close to pull-in, and, then, by averaging the results.

In spite of the nominally identical geometry of all the devices featuring the same beam length, Figure 3 testifies a large scattering in the measured electromechanical responses, independently of the test configuration and of the beam length. Such scattering is assumed to be induced by uncertainties in the geometric and mechanical properties of the polysilicon film constituting the movable structure of the test device. As shown in the Supplementary Material for an exemplary case (RR test configuration, l= 20 μm), the dispersion cannot be instead related to measurement inaccuracies.

An assessment of the above uncertainties can provide a means to foresee bounds on the mechanical response of MEMS devices with a characteristic size of compliant structural components on the same order of an average grain size. At variance with our former studies [32,35], wherein an approximated analytical coarse-grain model of the moving structure was adopted, in what follows a finite element model of the same structure is detailed to better assess, from a statistical perspective, the sources of uncertainty leading to the measured scattered response.

## 3. Numerics: Finite Element Analysis Allowing for Stochastic Effects

To model the quasi-static behavior of the devices, the coupled electromechanical problem was numerically solved in ANSYS [37]. By accounting for the relatively small in-plane width of the cantilever beam (h=2 μm) in comparison with its out-of-plane thickness (t=22 μm), and by accounting also for the deformation induced by the capacitors placed around the rotor, only the in-plane motion of the moving structure was considered. Therefore, a two-dimensional (plane strain) model was adopted. The mechanical domain was discretized using six-node (quadratic) PLANE183 triangular elements with displacement degrees of freedom, whereas the electrical domain (consisting in the gaps between conductors) was discretized using three-node (linear) PLANE223 elements featuring both displacement and voltage degrees of freedom. Each silicon grain boundary was discretized with elements featuring a characteristic size on the order of 50–100 μm, which was already reported in [24] to provide accurate mesh-independent results for the deformation characteristics of film representative volumes. The air gap between stators and rotor was instead discretized using at least three elements across its thickness, to avoid excessive distortion of the mesh up to pull-in. Overall, such space discretization is expected not to affect the accuracy of the numerical solution. For the typical grain morphologies considered here, the resulting space discretizations amounted to about 30,000 elements in the electrical domain and to 25,000 or 50,000 elements in the mechanical domain, depending on the beam length, overall consisting of 60,000 or 75,000 nodes. Such numbers obviously vary from one morphology to another, since the discretization has to be conforming with all the grain boundaries. The aforementioned features of space discretizations were somehow kept low by adopting a coarse-grained discretization for the moving plate, which is displaced by actuation almost like a rigid body and so is marginally deformed. Geometric nonlinearities were appropriately accounted for: while the mechanical domain undergoes rather small variations of its configuration and therefore a linearized kinematics proves sufficient to describe its response, the narrow electrical domain gets largely distorted by the actuation, especially close to pull-in. Therefore, the applied voltage (either $V_{L}$ or $V_{R}$) was smoothly increased in a step-by-step fashion, the current configuration of the model was continuously updated to look for the equilibrium condition, and then to compute the capacitance change relevant to sensing. Fringe field effects were disregarded: according to [38], depending on the allowed rotation and lateral displacement of the rotor preceding pull-in (always featuring small tilting angles), fringe field gives a variation of the measured capacitance amounting up to 15–20%. As values of the capacitance change were experimentally measured and considered in the simulations, the actual effects of the fringe field to be monitored are those related to the variation of the geometry of the narrow electrical domains induced by actuation. Approximated computations, in accordance with the formulations reported in [39], allow for stating that such effect is expected to induce a variation of the capacitance change amounting to 3–4% at most.

**Figure 4:**
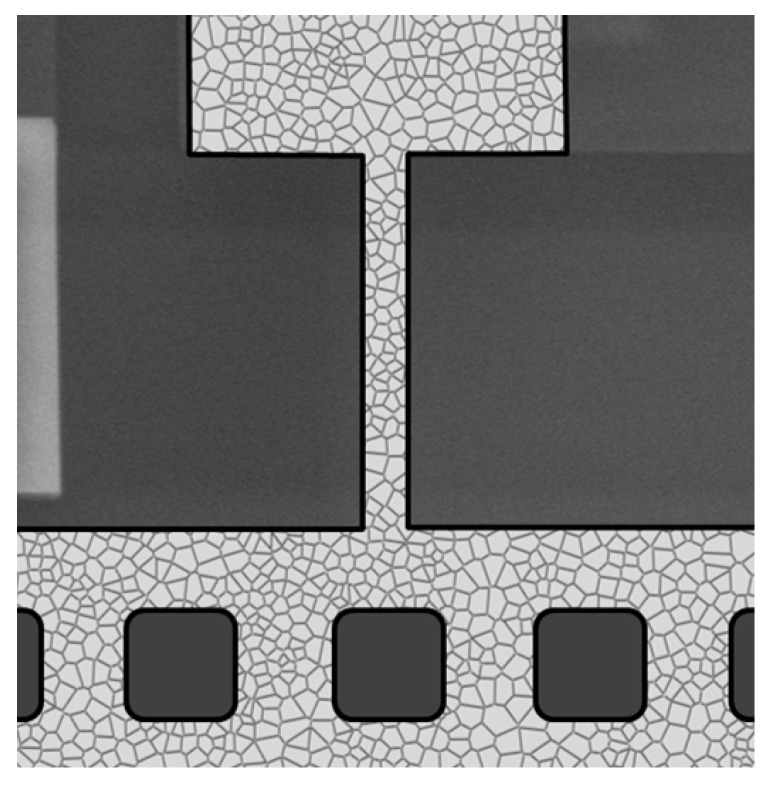
Example of the digital polysilicon morphology in the beam region.

Due to the small characteristic size of the tested polysilicon beam, the effects of lattice orientation of each single grain, and also of the orientation mismatch between adjacent grains are emphasized. Further to that, a local stress intensification along grain boundaries close to the anchor or to the connection between the beam and the plate, may also induce some reliability issues (see [40,41]). To allow for all these micromechanical effects, Monte Carlo simulations were adopted for all the test configurations: every simulation consists of a series of finite element analyses, each one characterized by a polysilicon morphology and a film geometry affected by over-etch. The two-dimensional realizations of the film morphology were obtained with a mildly-regularized artificial Voronoi tessellation of the whole plane (see Figure 4). The average in-plane size of crystal grains, thought as the radius of the relevant inscribed circle, was assumed to be 0.5 μm; the statistical distribution around this value was considered to be Gaussian, with a standard deviation of 0.1 μm. Each fcc silicon grain was therefore characterized by its own shape and also by the orientation of its crystal lattice (see [24,25]).

Due to the columnar structure of the film, see [24,34,42], one crystal orientation was always assumed to be aligned with the out-of-plane direction, i.e., perpendicular to the considered planar film; the other two crystal orientations were instead randomly varied in the plane, sampling the values out of a uniform probability distribution function in the range [0° 360°). Such specific features of the polysilicon film were not considered in our previous works [32,35], where a Bernoulli–Euler kinematics and homogenized elastic properties were instead adopted for the cantilever.

Silicon grains were modelled as orthotropic elastic bodies, with an in-plane stress–strain relationship governed by a stiffness matrix c that reads, in a local reference frame aligned with the two in-plane lattice orientations:

(1) $$c=\left[\begin{array}{ccc} c_{1111} & c_{1122} & 0\\ c_{1122} & c_{1111} & 0\\ 0 & 0 & c_{1212} \end{array}\right],$$

where $c_{1111}=165.7$ GPa, $c_{1122}=63.9$ GPa and $c_{1212}=79.6$ GPa (see [15,43]).

In [44], we proved that the variability of the effective cantilever stiffness induced by the polysilicon film morphology does not allow for catching the wide scattering in the device response depicted in Figure 3. Among the further uncertainty sources, the two main ones related to the mechanics of the movable structure are: the geometric imperfections, parametrized e.g., through the over-etch *O* [35], and the process-induced residual stresses and stress gradients, possibly giving rise to an initially displaced and/or rotated position of the rotor, even under no actuation. This latter source was investigated in [32,36] and it appeared to be less important than the former one, at least concerning the specific set-up geometry studied here. In fact, being the movable structure purposely designed to be statically determinate, the effects of residual stress components is reduced to a minimum, if not to zero; stress gradients provide instead a higher-order effect, which is here assumed negligible for the considered production process.

As far as the over-etch is concerned, it cannot be dealt with as a deterministic parameter at the micro-scale. Around the target value considered at the design stage, which was assumed to be zero for ease of discussion, a variability needs to be assumed to feed the Monte Carlo analyses and so define the actual geometry of each single structure. In the simulations, we considered for the over-etch a uniform probability distribution within the interval −150 nm ≤O≤ 150 nm, in accordance with the process specifications. Within each finite element analysis, we assumed a constant isotropic over-etch, over all the edges of the movable structure, sampled out from the mentioned uniform distribution; straight segments thus kept their shape independently of their orientation in the plane. In plane anisotropy of the over-etch would add additional variables to the statistical analysis here provided; moreover, for polycrystalline films, the vertical to lateral anisotropy is mainly of interest (see, e.g., [45]), but it turns out to be irrelevant for the present case of in-plane motion of the rotor.

While the geometric uncertainties strictly depend on the fabrication process and their extent can be reduced by process enhancements, the properties of the polysilicon film can be only bounded by the extreme values relevant to the single crystal case, i.e., according to [24,46]: Young’s modulus 130 GPa ≤E≤169 GPa; shear modulus 50.9 GPa ≤G≤79.6 GPa. In relation to the interpretation of the scattering of the *C*–*V* curves, independently of the beam length and of the actuation/sensing configuration, some reference results linked to such bounding properties are collected in Figure 3. The numerical (black) curves were obtained by setting *O* to be deterministically zero, in order to assess the effects of the spreading of the overall stiffness of the beam on the device response. Two different lattice orientations have been fictitiously considered for all the grains: a stiff one, with the lattice orientation [110] aligned with the longitudinal axis of the beam (the vertical direction in Figure 1) and a compliant one, with the lattice orientation [100] aligned with the beam axis. Further to that and in agreement with a bulk of literature wherein polysilicon is handled as an isotropic medium or, to be more specific, as a transversely isotropic material, results are also shown as provided by the following properties: Young’s modulus E=149.3 GPa, Poisson’s ratio ν= 0.172. These values have to be considered as linked to an asymptotic solution referring to a number of grains in a representative sample of the film growing to infinity. They were computed as the average between the upper and the lower bounds on them, as provided by the numerical homogenization procedure discussed in [24]. Accordingly, they do not depend on the grain size, which is a fluctuating value for each film morphology.

The plots show that the isotropic case can catch the trend of all the reported experimental curves. However, two main issues have to be reported: the isotropic response is sometimes biased away from the average experimental data (although not explicitly reported in the graphs, since the focus is here more on the scattering rather than on average features only); experimental data spans a region of the *C*–*V* plane that cannot be fully covered by the numerical results, with some plots falling out of the aforementioned bounds. This simple experimental/numerical comparison clearly shows that the experimental scattering cannot be explained only by allowing for the variation of the elastic moduli of the polysilicon film. In the next section, results are reported for the probabilistic study, wherein both mechanical and geometrical uncertainties were fully accounted for.

## 4. Discussion on the Statistics of Scattering

In what follows, the experimental results are compared with the outcomes of the Monte Carlo analyses described in Section 3. To guarantee convergence of the statistics, 200 simulations were carried out for each test configuration and for each beam length. Due to the limited number of experimental curves (10 for each beam length and test configuration), the statistics of scattering are represented in the form of a cumulative distribution function (CDF) of the capacitance change at a given value of the actuation voltage. Since the *C*–*V* plots smoothly evolve by increasing the value of $V_{R}$ or $V_{L}$, the voltage value for each case was selected as large enough to reduce the pollution induced by the measurement noise, hence approaching the pull-in for the more compliant structures.

**Figure 5:**
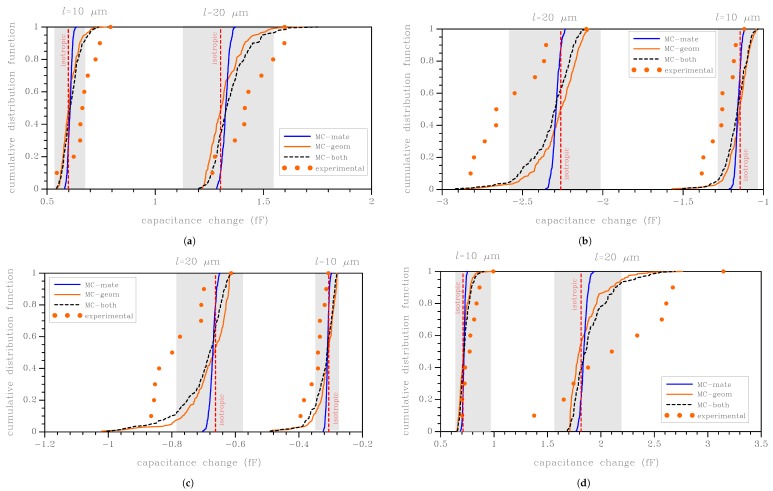
Comparison between numerical and experimental cumulative distribution functions: (**a**) RR; (**b**) RL; (**c**) LR; and (**d**) LL test configurations.

In Figure 5, the results for the four test configurations and for the two beam lengths l=10 μm and l= 20 μm are collected. Having adopted $V_{R}$ = 30 V and $V_{L}$ = 15 V as reference values for the actuation voltage, the outcomes of the Monte Carlo analyses are reported in terms of the CDF of the capacitance change induced by both the uncertainty sources (dashed black line), and also in terms of the CDF related only to the polysilicon morphology or to the over-etch (continuous blue and orange lines, respectively). These two latter data sets are provided to assess their relative contributions to the overall scattering of the data. In all the graphs, the intervals between the stiff and compliant bounds introduced in Section 3 are provided as shaded regions; the responses relevant to the isotropic case are reported too.

**Figure 6:**
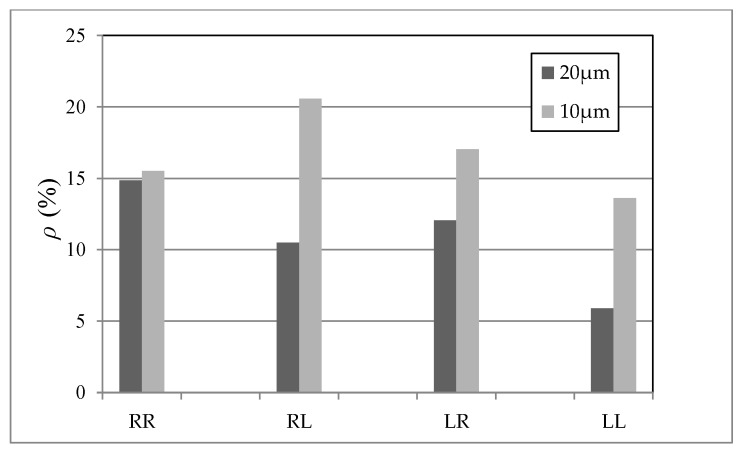
Effect of polysilicon morphology (only) on the scattering of the device response, measured through the ratio ρ between the standard deviations of numerical and experimental data at varying test configurations.

Starting from the discussion on material uncertainty, as already observed for Figure 3, the bounds on the elastic properties of polysilicon are shown to cover most of the measured data, although the percentage of the experimental results falling in the shaded regions depends on the beam length and on the test configuration. In comparison to these shaded regions, in all the cases, the experimental data are moved away towards the more compliant side. Such more compliant responses are characterized by larger amplitudes of the capacitance change under the given actuation voltage: for the RR and LL configurations, characterized by positive values of the capacitance change, some experimental results are out-of-bounds on the right side; for the RL and LR configurations, characterized instead by negative values of the capacitance change, some experimental results out-of-bounds are moved to the left side. By keeping in mind that the considered bounding values for the elastic properties of polysilicon are unrealistic in practice, it emerges that over-etch plays a significant role as far as the statistics of scattering are concerned. This aspect is further testified by the results related to the scattering induced by the mechanical properties only, with the over-etch always deterministically set to zero: the relevant CDFs all appear too steep, and they are not able to capture the experimental dispersion. Although the ratio between the in-plane cantilever width and the characteristic size of silicon grains is rather small (on the order of 2–3 in the present case, see Figure 4), the overall response is marginally affected by the film morphology, due to the beam slenderness. Such result is in accordance with the length-scale separation principle of the theory of homogenization, and also with the results reported in [24,25]. Although small, the effect of material heterogeneity on the foreseen scattering is reported in Figure 6, in terms of the ratio ρ between the numerical and experimental standard deviations, for the given applied voltage (either $V_{R}$ = 30 V or $V_{L}$ = 15 V). As the beam length *l* is decreased, ρ increases, though at different rates for the different test configurations: hence, the morphology effect becomes more relevant as the structural size decreases. This result is again expected, since, with a reduction of *l*, the ratio length/grain size decreases as well, and therefore the effect of variability of the microstructure gets enhanced. This observation thus shows one intrinsic hurdle in the miniaturization of MEMS structural components made of polysilicon.

Looking at the effect of over-etch on the results, we have to recall that relatively small changes of the beam width (nominally h= 2 μm) largely affect the beam moment of inertia, which is proportional to the real width cubed. On the electrostatic side, by increasing the width of the narrow capacitance gaps, the actuation and the capacitance measured in the deformed configuration are nonlinearly affected. Therefore, when uncertainties related to the geometry are considered (continuous orange lines), the corresponding CDFs in Figure 5 are less steep than those induced by material uncertainties. By comparing the present results with the experiments, for all the configurations but the LL-20 μm one, see Figure 5d, the geometrical uncertainties describe reasonably well the slope of the experimental CDFs. Nevertheless, the numerical CDFs look slightly translated along the horizontal axis with respect to the experimental ones.

If both the uncertainty sources are taken into account, as shown by the dashed black lines in Figure 5, the numerical CDFs move in the right direction towards the experimental data, although the model is still partially unable to perfectly match the experiments. A better agreement between the experimental data and the outcomes of the Monte Carlo analyses would be possible by exploiting e.g., strategies to enrich the numerical model in order to allow for inhomogeneous values of the over-etch. Other geometrical effects, like e.g., imperfections at the anchor and residual stress gradient-induced initial offsets of the rotor, can provide a further scattering effect. All these topics are beyond the scope of the current investigation and will be considered in future works (see also [32,35]).

## 5. Conclusions

In view of the current trend towards MEMS miniaturization, we investigated the influence of polysilicon grain morphology and geometrical uncertainties (induced by the over-etch) on the overall electromechanical response of purposely designed on-chip MEMS testing devices. The experimental data were compared with the results of finite element simulations. To get insights into the scattering shown by the experimental response at varying test configuration and geometry of the movable structure, Monte Carlo analyses were carried out. In the analyses, each polycrystalline morphology of the silicon film was digitally obtained via a Voronoi tessellation, and the over-etch was assumed isotropic and homogeneous all over the geometry borders.

As for what concerns the scattering in the system response, cumulative probability distribution functions of experimental and numerical capacitance changes at a given applied voltage were compared. Such comparison allowed for highlighting the following results:

- the polysilicon morphology has a non-negligible but relatively small effect on the overall response of the device, which becomes more relevant as the structural dimensions shrink;
- the statistics of over-etch, i.e., of the variation of the fabricated layout against the target one, need to be allowed for to better describe the scatterings observed in the experimental data;
- an offset between simulated and experimental statistics is still present because of other sources of uncertainty at the micro-scale not considered in this study, such as anchor effects or electric fringe fields.

The experimental evidence of this work pointed out the difficulties of miniaturizing polysilicon MEMS components towards dimensions close to the silicon grain size, still maintaining a robust functionality. Accordingly, future developments will focus on extending the experimental campaign with more test geometries, and on enriching the numerical model to account for the statistical effects induced by additional micromechanical imperfections.

## Supplementary Materials

The following is available online at http://www.mdpi.com/2072-666X/9/2/53/s1, Figure S1: *l* = 20 μm, RR test configuration; effect of measurement noise on the dispersion (represented through a box-plot) of the experimental response of one device.
