# Supplementary material for: Statistical Investigation of the Mechanical and Geometrical Properties of Polysilicon Films through On-Chip Tests
Source: Micromachines (Basel). 2018 Jan 30;9(2):53. doi: 10.3390/mi9020053 (PMC6187481; doi:10.3390/mi9020053)
Supplement: Supplementary file 1 [file micromachines-09-00053-s001.pdf]

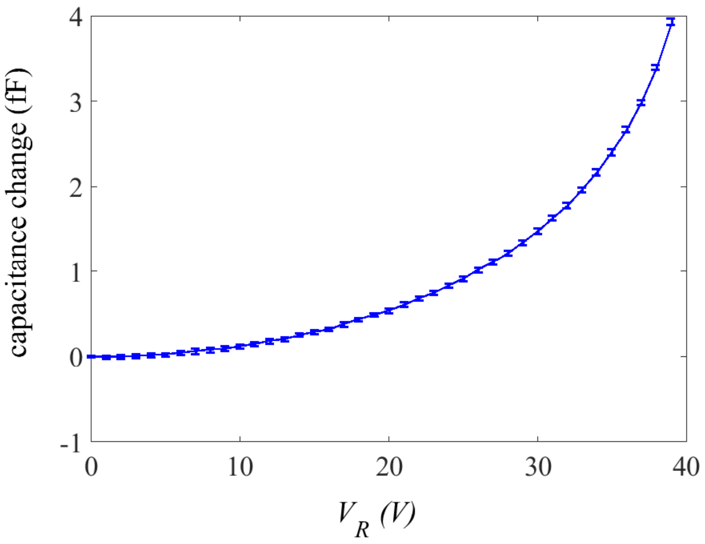

Figure S1. l=20 μm, RR test configuration; effect of measurement noise on the dispersion (represented through a box-plot) of the experimental response of one device. This plot implies that the measured scattered device responses are primarily governed by the dispersion of the parameters under investigation, and not by measurement errors, see also reference [34] cited in the paper.
